# MiDAS 2.0: an ecosystem-specific taxonomy and online database for the organisms of wastewater treatment systems expanded for anaerobic digester groups

**DOI:** 10.1093/database/bax016

**Published:** 2017-03-18

**Authors:** Simon Jon McIlroy, Rasmus Hansen Kirkegaard, Bianca McIlroy, Marta Nierychlo, Jannie Munk Kristensen, Søren Michael Karst, Mads Albertsen, Per Halkjær Nielsen

**Affiliations:** Department of Chemistry and Bioscience, Center for Microbial Communities, Aalborg University, Aalborg DK-9220, Denmark

## Abstract

Wastewater is increasingly viewed as a resource, with anaerobic digester technology being routinely implemented for biogas production. Characterising the microbial communities involved in wastewater treatment facilities and their anaerobic digesters is considered key to their optimal design and operation. Amplicon sequencing of the 16S rRNA gene allows high-throughput monitoring of these systems. The MiDAS field guide is a public resource providing amplicon sequencing protocols and an ecosystem-specific taxonomic database optimized for use with wastewater treatment facility samples. The curated taxonomy endeavours to provide a genus-level-classification for abundant phylotypes and the online field guide links this identity to published information regarding their ecology, function and distribution. This article describes the expansion of the database resources to cover the organisms of the anaerobic digester systems fed primary sludge and surplus activated sludge. The updated database includes descriptions of the abundant genus-level-taxa in influent wastewater, activated sludge and anaerobic digesters. Abundance information is also included to allow assessment of the role of emigration in the ecology of each phylotype. MiDAS is intended as a collaborative resource for the progression of research into the ecology of wastewater treatment, by providing a public repository for knowledge that is accessible to all interested in these biotechnologically important systems.

**Database URL:** http://www.midasfieldguide.org

## Introduction

Wastewater treatment is one of the largest biotechnological industries in the world. Wastewater itself is increasingly viewed as a resource with a growing focus on the recovery of inorganic resources, such as nitrogen and phosphorus, and bioenergy. As such, anaerobic digester technology is increasingly being implemented at full-scale treatment plants, primarily for the conversion of solid waste to methane gas for energy, giving the potential for net-energy production from wastewater treatment (1). As with activated sludge systems, anaerobic digestion is mediated by complex microbial communities, with the function, stability and efficiency reliant on the tightly coupled synergistic activities of specialized microbial sub-populations. As such, an understanding of the ecology of these systems will be important for optimal process design and operation (2,3).

Recent advances in sequencing technology now make high throughput analyses of complex microbial communities with 16S rRNA gene sequencing possible. This allows high-resolution monitoring over time, permitting correlation analyses with operational parameters for the identification of key phylotypes in full-scale wastewater treatment and biogas systems (3,4). Of subsequent importance to our understanding of these systems is the ability to estimate the function of these phylotypes, which becomes unreliable for classification at taxonomic levels higher than the species or genus (5). Classification of 16S rRNA gene sequences is mediated by curated public databases: including Silva, the Ribosomal Databases Project (RDP) and Greengenes (6–8). However, a large proportion of communities in wastewater treatment and biogas systems are uncultured and unclassified in these databases (9–14). The onerous task of manual taxonomic annotation has led to the development of ecosystem-specific taxonomies where annotation efforts are focussed on abundant and pertinent groups (15–18)—including the MiDAS database for the organisms of activated sludge (4). The MiDAS initiative initially provided a taxonomic database curated for abundant and process important phylotypes for activated sludge wastewater treatment systems with biological nutrient removal. It also incorporated a referenced online database that allows phylotype identity to be linked to information about the morphology, ecophysiology, abundance and distribution of genus members in full-scale treatment systems. The project was made feasible by the observation that relatively few genera make up the majority of organisms in full-scale wastewater treatment systems (12). Surveys of anaerobic digesters located at wastewater treatment plants reveal that these microbial communities also have a common set of abundant genera (9,13).

In this report, we present the expansion of the MiDAS field guide to include the organisms of the anaerobic digestion community. This includes an updated taxonomy and online profiles for the abundant organisms of full-scale anaerobic digester systems. In addition, the most abundant influent wastewater organisms are also incorporated, allowing for an assessment of the influence of migration on described phylotypes, which is critical to understanding their ecology. Coverage of the influent wastewater, activated sludge and anaerobic digester communities gives a holistic view of the microbial ecology of wastewater treatment facilities.

## Curation and expansion of the MiDAS taxonomy

The MiDAS taxonomy is a curated version of the SILVA taxonomy (7), with MiDAS release 2.1 based on SILVA release 1.23. As with the previous MiDAS release (4), the annotation of novel sequence clades was guided by the position of representative OTU sequences, added to the SILVA base tree with the ‘ARB parsimony insertion tool,’ and their closest full-length sequences (percentage sequence similarity). These amplicons represent the abundant organisms from large-scale surveys of influent wastewater, activated sludge and anaerobic digesters fed with primary sludge and surplus activated sludge (summarized in Table 1). There are further plans to incorporate anaerobic digester systems treating other organics, such as food and industrial wastes. While the current MiDAS release is based solely on lists from Danish treatment systems, it will be updated to be relevant to plants globally in the near future. Preliminary data for activated sludge shows that the same abundant organisms appear to be found in systems globally, making the current database version already relevant for use with systems outside Denmark (Nierychlo, M., Nielsen, P.H. and others, unpublished).

**Table 1.** The sequence data sets used to guide the curation of the MiDAS v. 2.1 taxonomy

System	Number of WWTPs	Sampling period	Sampling frequency (per year)	16S amplicon region	Kingdom	No. OTUs used	Reference
Wastewater influent	14	3 months	6	V1-3	Bacteria	50	Kirkegaard *et al.* (19)
Activated sludge	13	2 years	2–6	V4	Bacteria	104^a^	Saunders *et al.* (12)
	20	8 years	4	V1-3	Bacteria	100	McIlroy *et al.* (4)
AD—mesophilic	14 (26 ADs)	6 years	≤4	V1-3	Bacteria	40	Kirkegaard *et al.* (19)
				V3-5	Archaea	20	Kirkegaard *et al*, (19)
AD—thermophilic	5 (7 ADs)	6 years	≤4	V1-3	Bacteria	40	Kirkegaard *et al.* (19)
				V3-5	Archaea	20	Kirkegaard *et al.* (19)
					Total	374	

a OTUs 94% sequence similarity cut-off value. All others use a cut-off of 97%.

**Figure 1.**
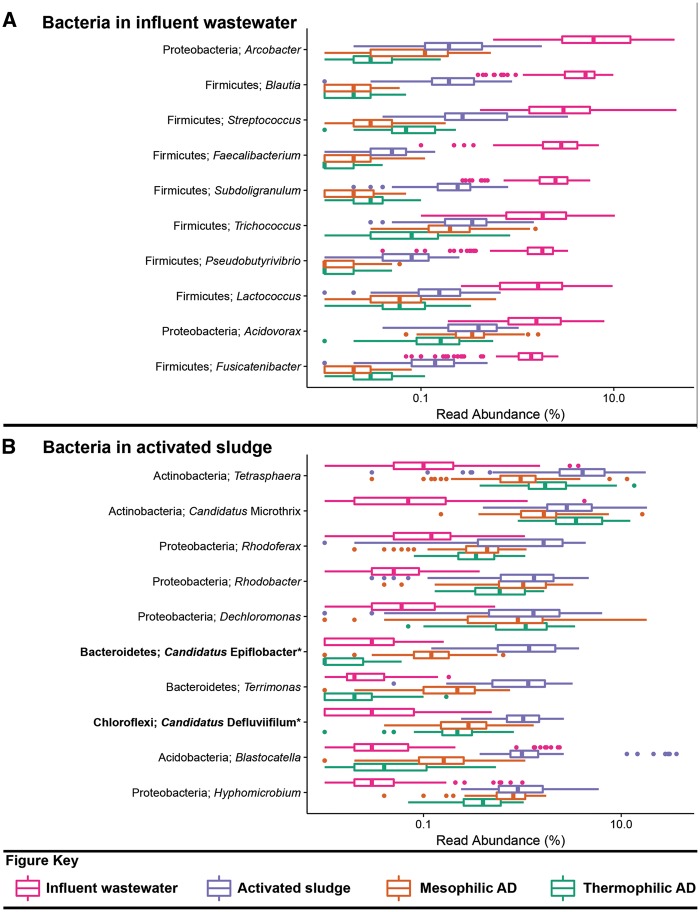
Box plot for the distribution of the 10 most abundant bacterial genus-level-taxa by median abundance in **A**. influent wastewater and **B**. activated sludge. Amplicon abundance values (V1-3 region) are given as a percentage of total bacterial reads for the influent wastewater, activated sludge and anaerobic digester communities (19). *Novel MiDAS genus level taxa are given in bold.

**Figure 2.**
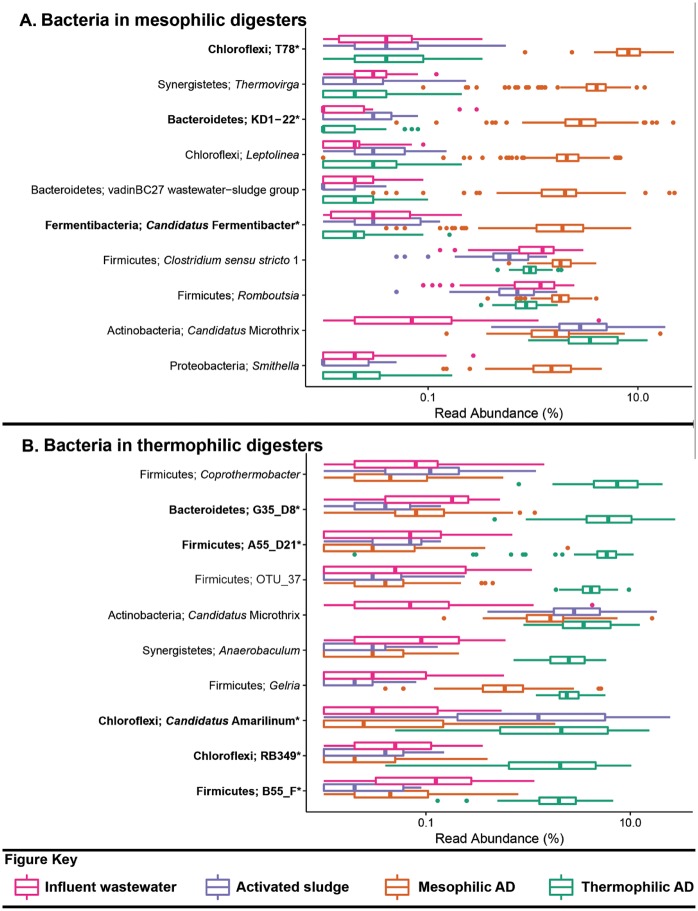
Box plot for the distribution of the 10 most abundant bacterial genus-level-taxa by median abundance in **A**. mesophilic and **B**. thermophilic anaerobic digesters. Amplicon abundance values (V1-3 region) are given as a percentage of total bacterial reads for the influent wastewater, activated sludge and anaerobic digester communities (19). *Novel MiDAS genus level taxa are given in bold.

**Figure 3.**
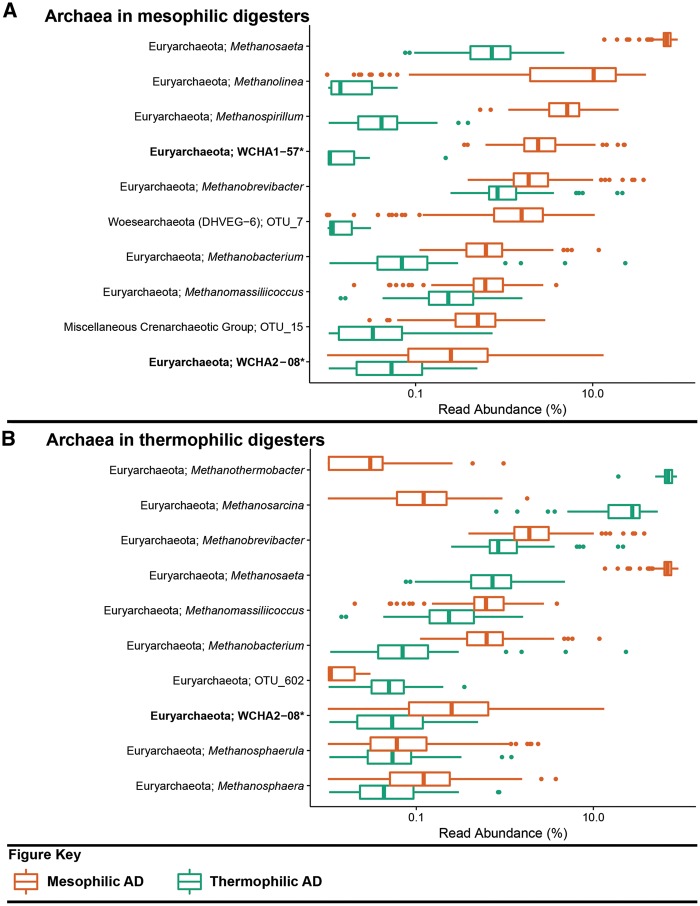
Box plot for the distribution of the 10 most abundant archaeal genus-level-taxa by median abundance in **A**. mesophilic and **B**. thermophilic anaerobic digesters. Amplicon abundance values (V3-5 region) are given as a percentage of total archaeal reads for each phylotype (19). *Novel MiDAS genus level taxa are given in bold.

The datasets used for the analyses presented in Table 2 and Figures 1–3 include influent wastewater (14 WWTPs, 3 months), activated sludge (24 WWTPs, 5 years), mesophilic ADs (26 reactors at 14 WWTPs, 6 years) and thermophilic ADs (7 reactors at 5 WWTPs, 6 years), and are taken from a recent 16S rRNA gene amplicon sequencing survey study of Danish WWTPs—the reader is referred to this article for further details (19). The benefit of applying the MiDAS taxonomy is evident in Table 2, which shows that a substantial proportion of the sequences present in the analysed systems are classified to novel MiDAS genus-level taxa. Several of the most abundant genera for the analysed sample types include novel MiDAS taxa (Figures 1–3)—such as T78, KD1-22 and *Candidatus* Fermentibacteria for the mesophilic ADs (Figure 2A). Importantly, these novel taxa would currently not be identified with other publically available taxonomies. The improvement for the different sample types varies, but on average it is > 25% for the Bacteria of anaerobic digesters. Limited improvement is shown for the influent genera, given most are associated with well-characterized faecal organism groups (Figure 1A;Table 2).

**Table 2.** Classification of wastewater treatment related systems with the MiDAS taxonomy

Environment	Kingdom	Amplicons classified to genus level^a^			
		Novel MiDAS taxa^b^		Total classified	
		% top 50 OTUs	% total reads	% top 50 OTUs	% total reads
Influent wastewater	Bacteria	0.0	1.2	94.0	89.0
Activated sludge	Bacteria	30.0	24.4	88.0	79.3
Mesophilic AD	Bacteria	38.0	26.6	84.0	79.6
	Archaea	12.0	4.3	88.0	87.0
Thermophilic AD	Bacteria	32.0	28.6	86.0	80.9
	Archaea	4.0	< 1	84.0	99.8

Sequences for all datasets (19) are classified with the MiDAS taxonomy v. 2.1.

a Sequences from all plants.

b Genus level taxa not included in the base SILVA 123 taxonomy.

Despite manual targeted annotation, not all of the abundant OTUs are assigned to a genus (Figures 2B and 3; Table 2). A lack of genus level annotation is partly due to the absence of closely related reference sequences in the database and the inadequate resolution of the short amplicon sequence for some closely related genera. This is particularly evident for placement of the archaeal sequences (see Figure 3), likely due to their relatively short length (variable region V3-5; 275 bps). The recently described method for high-throughput sequencing of the full rRNA SSU gene will improve the under-populated and environmentally skewed databases, resulting in improved amplicon classification (20). The ability to generate comprehensive datasets of full-length sequences, for a given environment, allows for the possibility for ecosystem-specific reference databases – which may also permit a higher resolution for amplicon classification.

## Linking taxonomy with function

**Figure 4.**
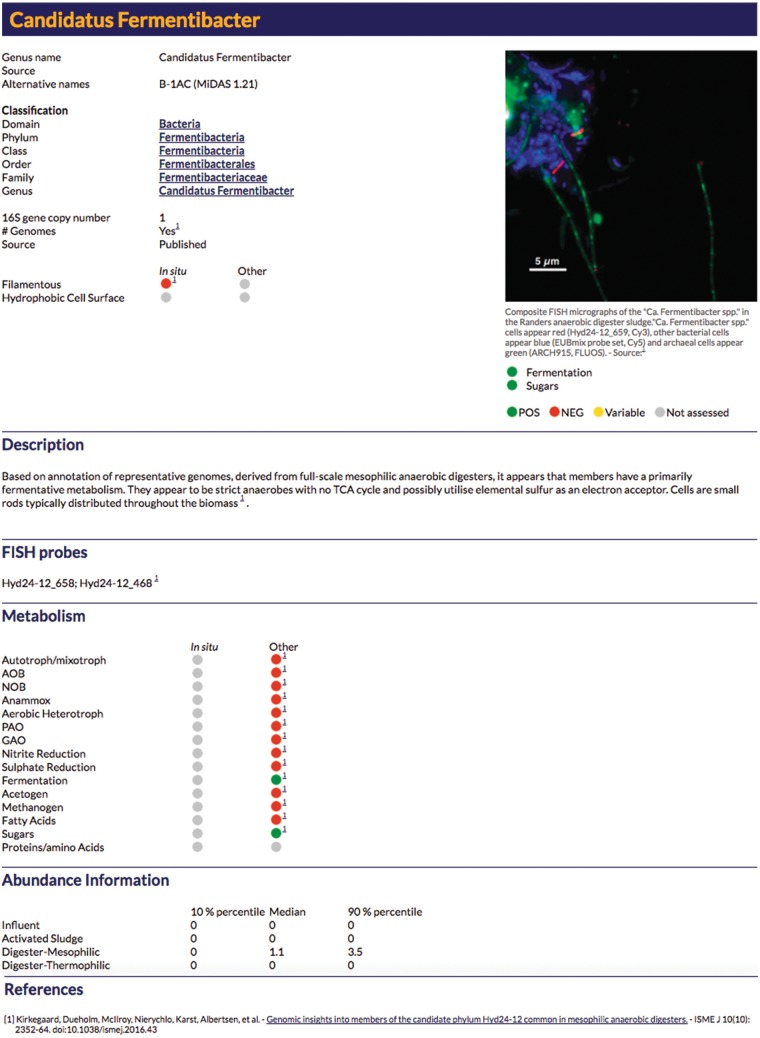
An example profile for the online MiDAS database. For a detailed explanation of each field see Supplementary Table S1.

The searchable online MiDAS database allows users to link genus level names, obtained with the use of the MiDAS taxonomy, to available information on their distribution and likely function in wastewater systems. Online MiDAS profiles are provided for the abundant genera of the influent wastewater, activated sludge and associated anaerobic digesters, based on extensive in-house surveys (Table 1; Figure 4). The current MiDAS release includes detailed profiles for 217 bacterial and 12 archaeal genera. These profiles are routinely updated and users are encouraged to contact us regarding relevant new information (mail@midasfieldguide.org).

**Figure 5.**
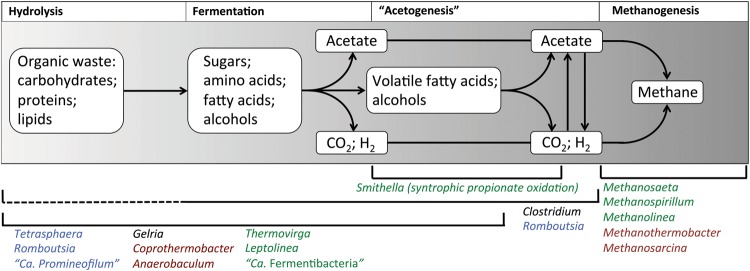
Basic outline of the carbon flow for the anaerobic digestion process. A selection of organisms found to be abundant in Danish systems that are associated with these process steps are listed for each (indicated by brackets, broken lines indicate uncertainty about the pathway in these organisms). Font colour indicates their niche system (green = mesophilic ADs; burgundy = thermophilic ADs; black = both mesophilic and thermophilic ADs; blue = abundant in both AS and recipient ADs).

Several fields relevant for the activated sludge system have been retained in the updated online database and key functional guilds for the anaerobic digester environment have been added. A full description of these fields is given in Supplementary Table S1. The key microbial mediated processes in the conversion of complex organics into methane include hydrolysis, fermentation, acetogenesis and methanogenesis (Figure 5). All except hydrolysis are covered with dedicated fields. Special roles in hydrolysis are noted in the organism *Description* fields; otherwise it is assumed that the fermentative organisms mediate the breakdown of complex organics. The *Acetogenesis* field in MiDAS uses the microbiological definition of the trait. Acetogenesis is routinely applied to broadly describe the synthesis of acetate. However, this definition is somewhat misleading as several metabolic strategies result in acetate production (21). The microbiological definition of acetogenesis is the conversion of CO_2_ and H_2_ to acetyl-CoA, which is utilized for biomass production or converted to acetate, via the reductive acetyl-CoA pathway (21).

Fields identifying problematic groups for the anaerobic digestion process are also included. Sulphate reducers are identified due to their unfavourable association with bio-corrosion of metal surfaces, odour production and competition with methanogens, resulting in reduced methane yield (22). In addition, hydrophobicity assessment has been included to identify organisms with a possible role in the stabilisation of foams. Foam formation is a common operational problem in both activated sludge systems and anaerobic digesters (23,24). Other pertinent information to the ecology and role of the MiDAS organisms is covered in the *Description* text field.

**Figure 6.**
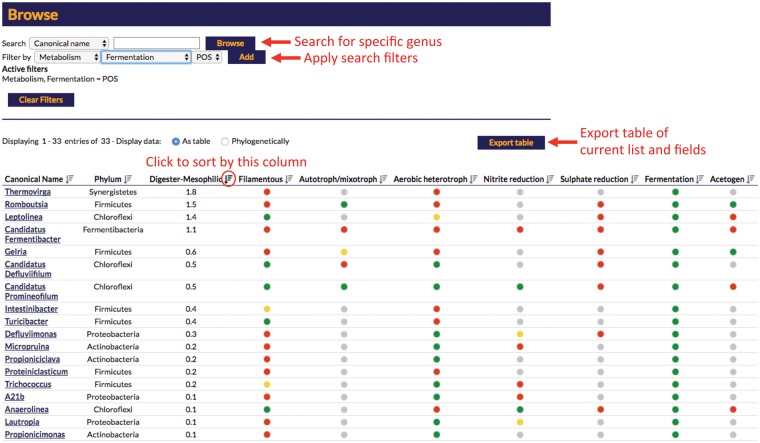
Screenshot of the result displayed from a search of the online MiDAS database. The displayed example is applying a search filter for fermentative organisms sorted by median read % abundance in mesophilic anaerobic digesters. Select features are highlighted in red.

In addition to accessing individual organism profiles, database searches can also be filtered by each of the profile fields, e.g. users can search for all genera with fermentative or filamentous members. Organism lists can also be sorted by these fields, e.g. by abundance in a particular system, and can also be exported in tabular format (Figure 6).

## Distribution and the importance of migration

An important expansion of MiDAS is the inclusion of abundance values of selected organisms for influent wastewater, activated sludge and anaerobic digesters. Viewing the abundance values in isolation ignores the important influence of migration (12). The population composition of the activated sludge and the anaerobic digesters is substantially influenced by the high migration with the primary sludge from the influent and secondary sludge [surplus activated sludge; Figures 1 and 2 (12,19,25)]. Focussing on the abundant members of the mesophilic sludge, the T78, *Thermovirga*, KD1-22, vadinBC27, *Leptolinea, Smithella* and ‘*Candidatus* Fermentibacter’ genera are all clearly selected for, while *Clostridium, Romboutsia* and ‘*Candidatus* Microthrix’ make up a substantial portion of the activated sludge fed into the system (Figure 2) and efforts to determine the activity of the latter are required. Migrating species may also be associated with operational problems, irrespective of metabolic activity, i.e. potential for stabilisation of foams for *Gordonia* spp. and ‘*Candidatus* Microthrix spp.’ (23).

## Concluding remarks

Recent advances in sequencing technology for the first time allow rapid and high throughput analyses of the composition of the microbial communities of wastewater treatment systems. However, there are a number of impediments to our progress in achieving a holistic understanding of the ecology of wastewater systems, which the MiDAS initiative endeavours to address. These include the inconsistent use of taxonomy, DNA extraction and primer selection, all of which have a substantial impact on the resulting analyses and make cross study comparison difficult (14,26,27). The MiDAS initiative consequently encourages the use of more consistent workflows by providing a public resource, which includes protocols and a taxonomy that are tailored to the wastewater treatment environment. An important subsequent point is the value of consistent genus-level-identifiers. These names enable the collation of relevant information for important phylotypes, which is also facilitated by the online MiDAS field guide. The ambition of MiDAS is to become a collaborative resource for those working in research and to facilitate the accumulation of knowledge made accessible to all with an interest in the biotechnological field of wastewater treatment and bioenergy.

## Supplementary data

Supplementary data are available at *Database* Online.

## Funding

This study was supported by the Danish Wastewater Association, Krüger A/S, Kemira A/S and approx. 50 municipal wastewater treatment plants (the ‘Microbial Database’), Aalborg University, the Innovation Fund Denmark (EcoDesign-MBR and NomiGas) [grant number 09-067230 and 1305-00018B] and, the Danish Council for Independent Research [grant no. 4093-00127A].

*Conflict of interest*. None declared.

## Supplementary Material

Supplementary Data
