# Supplementary material for: MiDAS 2.0: an ecosystem-specific taxonomy and online database for the organisms of wastewater treatment systems expanded for anaerobic digester groups
Source: Database (Oxford). 2017 Mar 18;2017:bax016. doi: 10.1093/database/bax016 (PMC5467571; doi:10.1093/database/bax016)
Supplement: Supplementary Data [file bax016_Supp.zip › xxxxMiDAS2_Table S1_Revision_Final.pdf]

Table S1: Field descriptions for online genus profiles

Property	Description
**Name**	A taxon's name status can be either: validly published, according to the International Code of Nomenclature of Bacteria (1,2); '*Candidatus*', for partially characterized organisms (3); or be an unpublished identifier given to uncultured phylotypes in the absence of an approved name.
**Source**	The original source of the genus name can be from the literature or be unique to the SILVA or MiDAS taxonomies. The latter relates to temporary identifiers for uncultured phylotypes.
**Alternative names**	Any antecedent names are included for each genus or species reclassified to the genus. Taxa merged or split in the MiDAS taxonomy are also noted.
**NCBI Taxon ID**	The taxon ID provides a reference to which all sequence files and projects, in the public NCBI databases, are linked (http://www.ncbi.nlm.nih.gov/taxonomy).
**16S gene copy number**	Putative 16S rRNA gene copy numbers are estimated from available genomes (https://rrndb.umms.med.umich.edu/). Such values should be considered in the interpretation of amplicon-sequencing data given that, relative to other taxa present, they influence the determined relative abundance (4).
**Genomes**	Stipulates whether or not a representative genome for the genus is available and provides selected reference(s) to relevant articles with genomic analyses.
Cell properties*	
**Filamentous morphology**	Excessive growth of organisms with a filamentous morphology has long been associated with the

	operational problem of 'bulking'. In this condition, interfloc bridging by the filaments impedes adequate settling of the biomass, resulting in loss of solids with the treated effluent (5,6).
**Hydrophobic cell surface**	Organisms with a hydrophobic surface have been associated with the stabilisation of foams in both activated sludge and anaerobic digester systems. Excessive build-up of surface foams can result in carryover of solids from clarifiers in wastewater treatment systems (6) and several operational problems in anaerobic digesters including biofouling of gas collection pipes and pumps (7).
Metabolism*	
**Autotroph/mixotroph**	Autotrophs are able to fix CO_2 as a carbon source for growth. Important examples in wastewater treatment include nitrite and ammonia oxidisers (NOB and AOB) and, anaerobic ammonia oxidisers (anammox). Mixotrophs can obtain carbon by fixing CO_2 and from organic sources (heterotrophy). The criteria for this MiDAS field is the potential for CO_2 fixation i.e. organisms possessing the Calvin-cycle.
**Ammonia oxidising bacteria (AOB)**	The AOB are chemolithotrophic organisms, which utilise ammonia as an energy source. Ammonia oxidation is coupled to the reduction of O_2 - which is the first step in the removal of nitrogen from waste streams. Ammonia is sourced from the ammonification of influent organics (8,9). It was recently revealed that some organisms are capable of oxidising ammonia all the way to nitrate – termed 'Comammox' (10,11).
**Nitrite oxidising bacteria (NOB)**	The NOB are chemolithotrophic organisms, which utilise nitrite as an energy source. Nitrite oxidation is coupled to the reduction of O_2 - which is the second step in the removal of nitrogen. Nitrite is

	obtained from the activities of the AOB (8,9).
**Anammox**	Anaerobic ammonium oxidising bacteria (Anammox) are chemolithotrophic organisms, which utilise ammonium as an energy source and nitrite as electron acceptor. Ammonium is oxidised to nitrite, which is subsequently reduced to dinitrogen gas, with CO_2 as their sole carbon source (12). Specialised processes enrich for these organisms for the anaerobic removal of ammonia from waste streams (8).
**Aerobic**	Organisms able to grow in the presence of oxygen, usually by using oxygen as electron acceptor.
**Polyphosphate accumulating organisms (PAO)**	The PAOs are enriched for in wastewater treatment systems configured for enhanced biological phosphorus removal (EBPR). They are responsible for the bulk of biological phosphorus removal from wastewater (13). The principle of EBPR systems relies on the cycling of biomass between carbon rich anaerobic conditions and carbon deficient aerobic conditions. The PAOs accumulate excess levels of phosphorus as polyphosphate under aerobic conditions, which they utilise as an energy source for substrate assimilation under anaerobic conditions. Thus wastage of polyphosphate rich biomass from the aerobic tank leads to net P removal from the system (13). Earlier definitions of the PAO included cycling of glycogen and polyhydroxyalkanoates (PHAs) in accordance with the model Accumulibacter PAO. However, the *Tetrasphaera* PAO do not accumulate PHA or cycle glycogen in the same way (14). As such, in MiDAS, the cycling of excess polyphosphate under the dynamic conditions of EBPR is applied to define a PAO.
**Glycogen accumulating**	The GAO are well adapted to the dynamic conditions of EBPR where they compete with the PAO for

**organisms (GAO)**	resources. Under anaerobic conditions they utilise aerobically stored glycogen for the energy and reducing equivalents required for carbon uptake and storage as PHAs. Anaerobically stored carbon is utilised under subsequent aerobic conditions for growth and replenishment of glycogen stores. As they do not cycle polyphosphate, but compete with the PAO for nutrient resources, their proliferation is thought to be at the expense of P removal efficiency in EBPR (13).
**Nitrite reducing bacteria (denitrifiers)**	The denitrifiers are usually facultative-anaerobic heterotrophic organisms utilising nitrate and/or nitrite as electron acceptors in the absence of oxygen. Denitrification is the final step of nitrogen removal from wastewater and generally occurs under anaerobic conditions. Nitrate is available from the oxidation of ammonia by the aerobic activities of the nitrifiers (AOB and NOB) (8). Denitrification involves the sequential reduction of nitrate to inert dinitrogen gas, via several intermediates (nitrite, nitric oxide (gas) and nitrous oxide (gas)) (9). Not all organisms involved in denitrification possess all the genes for reduction of nitrate all the way to dinitrogen gas. As such, for MiDAS profiles, the nitrite reducing organisms are selected to represent the denitrifiers; given denitrification is defined as "the dissimilatory transformation of nitrate or nitrite to a gas species concomitant with energy conservation" (9) and not all organisms capable of nitrate reduction can reduce nitrite (8).
**Sulphate reducing bacteria**	These organisms utilise sulphate as a terminal electron acceptor producing hydrogen sulphide. A variety of sulphate reducers have been described, being able to degrade a range of organic compounds to acetate or completely to CO_2. A variety of alternate electron acceptors have also been demonstrated, including Fe(III), nitrate and organics (fermentation) (15). Therefore, a high

	abundance does not always reflect high levels of sulphate reduction. They are thought not to degrade complex polymers and rely on the activities of the hydrolysers and fermenters for a supply of carbon. The abundance and activity of these organisms is not favourable in wastewater treatment and anaerobic digestion as they compete with the methanogens for substrate, giving a lower methane yield, and hydrogen sulphide is toxic, odorous and associated with bio-corrosion problems in sewer systems and treatment facilities (15).
**Fermenter**	During fermentation, organic compounds act as both the electron acceptor and donor. Organic molecules, such as sugars and amino acids, are utilised as the energy and carbon source. In the absence of, or the inability to utilise, external electron acceptors, organic molecules act as the electron acceptor to balance the redox of the cell. Common by-products include volatile fatty acids, alcohols, hydrogen and CO_2.
**Acetogen/syntrophic acetate oxidiser (SAO)**	Acetogenesis is routinely applied to broadly describe the biological synthesis of acetate. However, this definition is somewhat misleading as several metabolic strategies result in acetate production (16). MiDAS therefore applies the strict microbiological definition. True acetogens employ the reductive acetyl-CoA pathway for the synthesis of acetyl-CoA that can be converted to acetate or utilised as a source of carbon for biomass production. In this pathway, hydrogen and CO_2 are utilised as an electron donor and acceptor, respectively (16). Acetogens often utilise alternative donors and acceptors to supplement or replace the use of the pathway for energy or carbon supply depending on growth conditions. Commonly, the pathway operates in conjunction with fermentative pathways,

	operating to replace generated reducing equivalents in the reduction of CO_2 (16). Importantly, organisms possessing the reductive acetyl-CoA pathway can reverse the pathway to oxidise acetate to CO_2 and H_2 in concert with hydrogenotrophic methanogens. This is known as syntrophic acetate oxidation (SAO) as it requires the methanogens to remove H_2 to make the pathway thermodynamically favourable (17).
**Methanogen**	The methanogens couple energy production to the generation of methane gas from a selected range of substrates – including H_2 and CO_2 (hydrogenotrophic methanogenesis), acetate (acetoclastic methanogenesis) and formate, as well as some single carbon (C1) compounds and alcohols. Known methanogens are confined to the domain Archaea and anoxic environments (18).
Substrate assimilation*	The three main classes of organics fed into anaerobic digester systems are represented here: *Lipids/fatty acids; carbohydrates/sugars; proteins/amino acids*. In this MiDAS field guide there is no differentiation between those able to use complex organics and those only able to utilise simple compounds released during hydrolysis. Where known, this is covered in the genus description. Organisms only need to reportedly assimilate one compound for the class to be designated as positive for this field.
Abundance information	This data is taken from large scale 16S rRNA gene amplicon sequencing surveys of Danish WWTPs.
**Influent**	Influent wastewater taken from 14 plants over 3 months (20).
**Activated sludge**	20 full-scale WWTPs over 8 years (19).
**Mesophilic anaerobic digester**	26 digesters at 13 full-scale WWTPs over 6 years (20).

**Thermophilic anaerobic digester**	7 digesters at 5 full-scale WWTPs over 6 years (20).
**Predominant in**	Organisms can be abundant in, and perhaps active, in multiple systems, but this indicates the environment in which they appear to be most suited/successful. These include the influent, activated sludge, mesophilic ADs and thermophilic ADs.
**Description**	A more detailed summary of what is known about the organism with a focus on its physiology.
**Diversity**	Known diversity within the genus, such as clades/subgroups and species, are given here. For some genera, subgroups with varied phenotypic properties are reported.
**Distribution plant/configuration**	Known correlations to plant configurations or operational parameters.
**Fluorescence *in situ* hybridisation (FISH) probes**	Available FISH probes for *in situ* detection of the genus and/or its sub-groups.
**Taxonomy**	Classification is given at 6 taxonomic levels: Kingdom; Phylum; Class; Order; Family; Genus

*The evidence for *in situ* traits is separated from pure culture and genomic information (collectively represented by the field 'other evidence'); given the latter two represent only the potential of the organism for a phenotypic trait *in situ*. Isolates and available genomes may not represent species *in situ* (21,22). In addition, organisms are known to behave differently in complex communities and may not use the pathways they have the potential for (23). Therefore, *in situ* evidence is more informative. If available, *in situ* evidence is displayed during organism searches. If not available then other evidence is displayed instead.

References

1. Lapage, S., Sneath, P., Lessel, E., *et al.* (1992) *Rules of Nomenclature with Recommendations*. ASM Press.
2. Whitman, W. B. (2016) Modest proposals to expand the type material for naming of prokaryotes. *Int. J. Syst. Evol. Microbiol.*, **66**, 2108–12.
3. Murray, R. G. and Stackebrandt, E. (1995) Taxonomic note: implementation of the provisional status Candidatus for incompletely described procaryotes. *Int. J. Syst. Bacteriol.*, **45**, 186–7.
4. Stoddard, S. F., Smith, B. J., Hein, R., *et al.* (2014) rrnDB: improved tools for interpreting rRNA gene abundance in bacteria and archaea and a new foundation for future development. *Nucleic Acids Res.*, **43**, D593-598.
5. Nielsen, P. H., Kragelund, C., Seviour, R. J., *et al.* (2009) Identity and ecophysiology of filamentous bacteria in activated sludge. *FEMS Microbiol Rev*, **33**, 969–998.
6. Jenkins, D., Richard, M. G. and Daigger, G. T. (2004) *Manual on the Causes and Control of Activated Sludge Bulking, Foaming and Other Solids Separation Problems*; 3 rd. edition; CRC Press LLC: London, England.
7. Ganidi, N., Tyrrel, S. and Cartmell, E. (2009) Anaerobic digestion foaming causes – A review. *Bioresour. Technol.*, **100**, 5546–5554.
8. Daims, H. and Wagner, M. (2010) The microbiology of nitrogen removal. In: *Microbial ecology of activated sludge*; Seviour, R. J.; Nielsen, P. H., Eds.; IWA Publishing: London, 2010; pp. 259–280.
9. Zumft, W. G. (1997) Cell biology and molecular basis of denitrification. *Microbiol. Mol. Biol. Rev.*, **61**, 533–616.
10. Daims, H., Lebedeva, E. V., Pjevac, P., *et al.* (2015) Complete nitrification by *Nitrospira* bacteria. *Nature*, **528**, 504-9.
11. van Kessel, M. A. H. J., Speth, D. R., Albertsen, M., *et al.* (2015) Complete nitrification by a single microorganism. *Nature*, **528**, 555-9.

12. Jetten, M. S. M., Niftrik, L. van, Strous, M., *et al.* (2009) Biochemistry and molecular biology of anammox bacteria. *Crit. Rev. Biochem. Mol. Biol.* **44.** 65-84.

13. Oehmen, A., Lemos, P. C., Carvalho, G., *et al.* (2007) Advances in enhanced biological phosphorus removal: from micro to macro scale. *Water Res.*, **41**, 2271–2300.

14. Kristiansen, R., Nguyen, H. T. T., Saunders, A. M., *et al.* (2013) A metabolic model for members of the genus *Tetrasphaera* involved in enhanced biological phosphorus removal. *ISME J.*, **7**, 543–54.

15. Muyzer, G. and Stams, A. J. M. (2008) The ecology and biotechnology of sulphate-reducing bacteria. *Nat. Rev. Microbiol.*, **6**, 441.

16. Drake, H. L., Küsel, K. and Matthies, C. (2013) Acetogenic Prokaryotes. In *The Prokaryotes - Prokaryotic Physiology and Biochemistry*; Rosenberg, E.; De Long, E. F.; Lory, S.; Stackebrandt, E.; Thomson, F., Eds.; Springer-Verlag: Berlin; pp. 3–60.

17. Hattori, S. (2008) Syntrophic Acetate-Oxidizing Microbes in Methanogenic Environments. *Microbes Environ.*, **23**, 118–127.

18. Whitman, W. B., Bowen, T. L. and Boone, D. R. (2014) The methanogenic bacteria. In: *The Prokaryotes*; Rosenberg, E.; De Long, E.; Lory, S.; Stackebrandt, E.; Thomson, F., Eds.; Springer Berlin Heidelberg: Berlin, Heidelberg; pp. 123–163.

19. McIlroy, S. J., Saunders, A. M., Albertsen, M., *et al.* (2015) MiDAS: the field guide to the microbes of activated sludge. *Database*, **2015**, bav062.

20. Kirkegaard, R.H., McIlroy, S.J., Kristensen, J.M., *et al.* (2017) Identifying the abundant and active microorganisms common to full-scale anaerobic digesters. *bioRxiv*. doi: doi: https://doi.org/10.1101/104620.

21. Albertsen, M., Hansen, L.B., Saunders, A.M. *et al.* (2012) A metagenome of a full-scale microbial community carrying out enhanced biological phosphorus removal. *ISME J.*, **6**, 1094–106.

22. Kindaichi, T., Nierychlo, M., Kragelund, C. *et al.* (2013) High and stable substrate specificities of microorganisms in enhanced

biological phosphorus removal plants. *Environ. Microbiol.*, **15**, 1821–31.

23. McIlroy, S.J., Lapidus, A., Thomsen, T.R. *et al.* (2015) High quality draft genome sequence of *Meganema perideroedes* str. GR1^{T} and a proposal for its reclassification to the family Meganemaceae fam. nov. *Stand. Genomic Sci.,* **10**, 23.
